# A Deep Learning Framework About Traffic Flow Forecasting for Urban Traffic Emission Monitoring System

**DOI:** 10.3389/fpubh.2021.804298

**Published:** 2022-01-25

**Authors:** Baozhen Yao, Ankun Ma, Rui Feng, Xiaopeng Shen, Mingheng Zhang, Yansheng Yao

**Affiliations:** ^1^State Key Laboratory of Structural Analysis for Industrial Equipment, School of Automotive Engineering, Dalian University of Technology, Dalian, China; ^2^CIECC Overseas Consulting Co., Ltd., Beijing, China; ^3^School of Mechanical and Electrical Engineering, Anhui Jianzhu University, Hefei, China

**Keywords:** urban traffic construction, traffic flow analysis, deep learning, graph, prediction model

## Abstract

As urban traffic pollution continues to increase, there is an urgent need to build traffic emission monitoring and forecasting system for the urban traffic construction. The traffic emission monitoring and forecasting system's core is the prediction of traffic emission's evolution. And the traffic flow prediction on the urban road network contributes greatly to the prediction of traffic emission's evolution. Due to the complex non-Euclidean topological structure of traffic networks and dynamic heterogeneous spatial-temporal correlations of traffic conditions, it is difficult to obtain satisfactory prediction results with less computation cost. To figure these issues out, a novel deep learning traffic flow forecasting framework is proposed in this paper, termed as Ensemble Attention based Graph Time Convolutional Networks (EAGTCN). More specifically, each component of our model contains two major blocks: (1) the global spatial patterns are captured by the spatial blocks which are fused by the Graph Convolution Network (GCN) and spatial ensemble attention layer; (2) the temporal patterns are captured by the temporal blocks which are composed by the Time Convolution Net (TCN) and temporal ensemble attention layers. Experiments on two real-world datasets demonstrate that our model obtains more accurate prediction results than the state-of-the-art baselines at less computation expense especially in the long-term prediction situation.

## Introduction

With the rapid development of urban traffic construction, traffic emission has attracted more and more attention from public. The traffic emission contains carbon monoxide, nitrogen oxides and particulate matter, which are the main causes of smog and photochemical smog pollution (1, 2). Public's health pays much price for the traffic emission (2–5).

As a result, there is a need for urban traffic construction to establish an effective environmental monitoring and early warning system (2), whose core is the accurate prediction of the evolution of traffic emission. The trend of traffic emission evolution is mainly affected by traffic conditions including traffic flow, traffic velocity and road occupancy (6). Predicting the emission of traffic road networks means predicting trends of those traffic condition variables (7). Therefore, the accurate and efficient predictions of traffic condition variables' trends can provide scientific foundation for predicting the evolution of urban traffic emission. The task of traffic condition variables' predictions is to provide punctual, continuous and precise traffic condition variables prediction information based on the past measurements of traffic and the underlying road networks. Among those traffic condition variables, traffic flow is harder to predict (8). The challenges of traffic flow prediction can be summarized into the following two parts: accuracy and efficiency (9).

First, the accuracy problem origins from non-Euclidean topological structure of traffic networks, the stochastic characteristics of the non-stationary traffic patterns and inherent difficulties in multiple steps ahead prediction (10). The key to improve the accuracy of prediction is to capture simultaneously the dynamic heterogeneous spatial-temporal correlations of traffic conditions. On the one hand, the spatial correlations can not only be found at a local scale but also in a wide range of the traffic networks. Two distant roads in a traffic network might have high correlations, too (11). And different locations have different impacts on the study location (12). Moreover, since traffic is constantly evolving, the spatial correlations are dynamic (10). For instance, in the morning, the correlations between a residential area and a business center could be strong; whereas in late evening, the correlations between those might be very weak (13). On the other hand, traffic observations exhibit autocorrelations at the adjacent time intervals and show cyclical patterns. Traffic observation variations are affected by vehicular traffic lights, changes in weather, and other factors (9). Some of these factors play a long-term decisive role, making the variations show specific trends and certain regularities, while others play a short-term role, introducing some uncertainty into the variations. And the temporal correlations and periodicity vary in different time-of-day (10), which brings great difficulties to the prediction of traffic flow. Secondly, The efficiency problems of predictions origin from the scale of traffic flow predictions. On the spatial parts, the large size of traffic networks requires more sensors to detect traffic status. On the temporal parts, the application of traffic flow predictions needs longer forecasting steps. Both two parts increase the scale of predictions, which will cost more time.

Historically, related field researchers have exploited statistical methods, machine learning and deep learning approaches for modeling the complex temporal-spatial patterns of traffic flow forecasting problems. The widely used statistical methods include autoregressive integrated moving average (ARIMA) (14–16), Kalman filtering (17), Markov chain (18), and exponential smoothing methods (19–21). Those classical statistical models were purely inductive methods, which placed strong stationary assumptions on the traffic flow sequence. However, it was difficult to satisfy these assumptions in the real world due to the inherent complexity of traffic data. Therefore, those classical statistical methods did not have enough capability to capture dynamic patterns of traffic flow. And each step prediction was based on the prior predictions, which led to the propagation and accumulation of errors.

Along with the development of the computing device and information explosion, machine learning models have caused wide public concerns, including k-nearest neighbors (22), support vector regression (23) and random forest (24). Complex nonlinear traffic data can be regressed by those ML methods, but the premises were to conduct detailed feature engineering, which was critical but difficult. Furthermore, the power of capturing the complex non-stationary temporal patterns was limited by ML models' shallow architectures, especially for long-term forecasting (10).

Deep Learning (DL) network is an effective tool for regression problems like the traffic flow forecasting. This method aims to automatically identify patterns and extract features from the historical information by constructing an appropriate parameter space. The DL models have made breakthroughs in many domains, such as speech recognition and image processing. Those progress made by DL has drawn substantial interests among transportation researchers and they have been trying to apply deep learning models in many traffic prediction problems. Initially, the traffic status data was simply treated as normal temporal sequence and was predicted by classical Recurrent Neural Networks (RNNs) like Long Short Term Memory (LSTM) and Gated Recurrent Unit neural networks (GRU) (25, 26). Those networks neglected the modeling of traffic data's spatial attributes. Subsequently, some related researchers began to take both the spatial patterns and temporal patterns into consideration. Convolutional Neural Networks (CNN) which took charge of extracting spatial dependencies from traffic data was introduced into RNNs. Du et al. proposed a hybrid deep learning framework which consists of CNN and RNNs for short-term traffic flow forecasting (27). The Fusion Convolutional Long Short Term Memory Network (FCL-Net) (28) proposed by Ke et al. stacked and fused multiple LSTM layers, standard LSTM layers and CNN layers to capture the spatial-temporal characteristics of explanatory variables. Shi et al. constructed the convolutional LSTM model by combining the normal fully-connected LSTM and convolutional layers (29). Those models just treated the traffic status data at a certain time slice as an image. CNN as a typical deep neural network can effectively capture the spatial features of grid data. However, due to the non-Euclidean topology of the traffic networks, CNN actually does not have enough ability to extract the spatial patterns of the road networks. Recently, the Graph Convolution Network (GCN) has been widely used because this network can generalize the traditional convolution operations to non-Euclidean graph structure data (30). Many forecasting models based on GCN were proposed. Li et al. proposed graph and attention-based long short-term memory network (GLA) which was composed of GCN and LSTM (31). Zhu et al. proposed a new traffic flow prediction method based on RNN-GCN and the Belief Rule Base (BRB) (32). Spatial Temporal Graph Convolutional Networks (STGCN) was proposed by Yu et al., which combined GCN and 1D-CNN to capture spatial-temporal patterns (5). Attention Based Spatial Temporal Graph Convolutional Networks (ASTGCN) (12) presented by Guo et al. confused STGCN with attention layers which were used to capture the dynamic spatial correlations among nodes relying only on traffic flow data. However, these GCN based methods still have many problems. On the one hand, those methods could not comprehensively capture spatial-temporal dynamic heterogeneous features of traffic data which have significant influence on the traffic forecasting issues. On the other hand, the parts of those models which were responsible to capture temporal patterns were constructed by either RNNs or normal 1D CNN. The models constructed by RNNs did well in long-term dependencies' capturing but required too much computing time and suffered from gradient exploding or vanishing (33). The models constructed by 1D CNN were able to decrease the computation expense but needed more stacked layers to capture long-term temporal dependencies. DL models with too deep convolution layers might lose some key information in long-term forecasting, which results in the decline of forecasting accuracy.

In the paper, a novel deep learning model named Ensemble Attention Graph Time Convolutional Networks (EAGTCN) is proposed to predict traffic flow in the road network dimension. This model can capture more comprehensive dynamic heterogeneous spatial-temporal features of traffic data effectively and efficiently. Significantly, the model is applicable to the other traffic condition variables' forecasting and provides the solid foundation for traffic emission's prediction and monitoring. The main contributions of this paper are summarized as follows:

1. We propose an ensemble attention mechanism which is able to dig out the global dynamic heterogeneous spatial-temporal correlations from traffic sequence.
2. TCN is applied to capture basic temporal dependencies. The TCN has much longer effective memory while can be trained fast.
3. Our model is evaluated with the real-world traffic data, showing that our model outperforms than the state-of-art, especially in the long-term prediction situation.

## Problem Description

### Traffic Network Based on Graph

Normally, a spatial-temporal graph is composed of nodes and edges which connect those nodes. Spatial-temporal graph is defined as ${\text{G}}^{\left(\text{τ}\right)}=\left({\text{V}}^{\left(\text{τ}\right)},{\text{E}}^{\left(\text{τ}\right)},{\text{V}}_{\text{attr}}^{\left(\text{τ}\right)},{\text{E}}_{\text{attr}}^{\left(\text{τ}\right)}\right)$, where τ denotes the time slice τ. ${\text{V}}^{\left(\text{τ}\right)}=\left\{{\text{V}}_{1},{\text{V}}_{2},{\text{V}}_{3},\cdots \;,{\text{V}}_{\text{j}}\right\}$ represents all the nodes of the graph at time slice τ. ${\text{E}}^{\left(\text{τ}\right)}=\left\{\left({\text{e}}_{1},{\text{r}}_{1},{\text{s}}_{1}\right),\left({\text{e}}_{2},{\text{r}}_{2},{\text{s}}_{2}\right),\cdots \;,\left({\text{e}}_{\text{k}},{\text{r}}_{\text{k}},{\text{s}}_{\text{k}}\right)\right\}$ denotes the whole edges of the graph at time slice τ. e_k_ = v_ij_ represents the edge k between node i and node j. r_k_, s_k_ denote the edge k's receiving node and sending node respectively. $V_{attr}^{\left(\tau \right)}\in R^{n\times c}$ represents the features of all nodes at time slice τ, where n = |V^(τ)^| denotes the number of nodes and c denotes the dimension of a node feature vector. ${\text{E}}_{\text{attr}}^{\left(\tau \right)}\in {\text{R}}^{\text{u}\times \text{d}}$ represents the features of all edges at time sliceτ, where u = |E^(τ)^| denotes the number of edges, and d denotes the dimension of a edge feature vector. The structure of graph G^(τ)^ can be represented by (V^(τ)^, E^(τ)^). The adjacency matrix $\text{A}=\left({\text{A}}_{\text{ij}}\right)\in {\text{R}}^{\text{n}\times \text{n}}$ can be transferred from (V^(τ)^, E^(τ)^), in which A_ij_ = 1 if there is an edge between node i and node j and A_ij_ = 0 otherwise (A_ii_ = 0 ).

**Figure 1:**
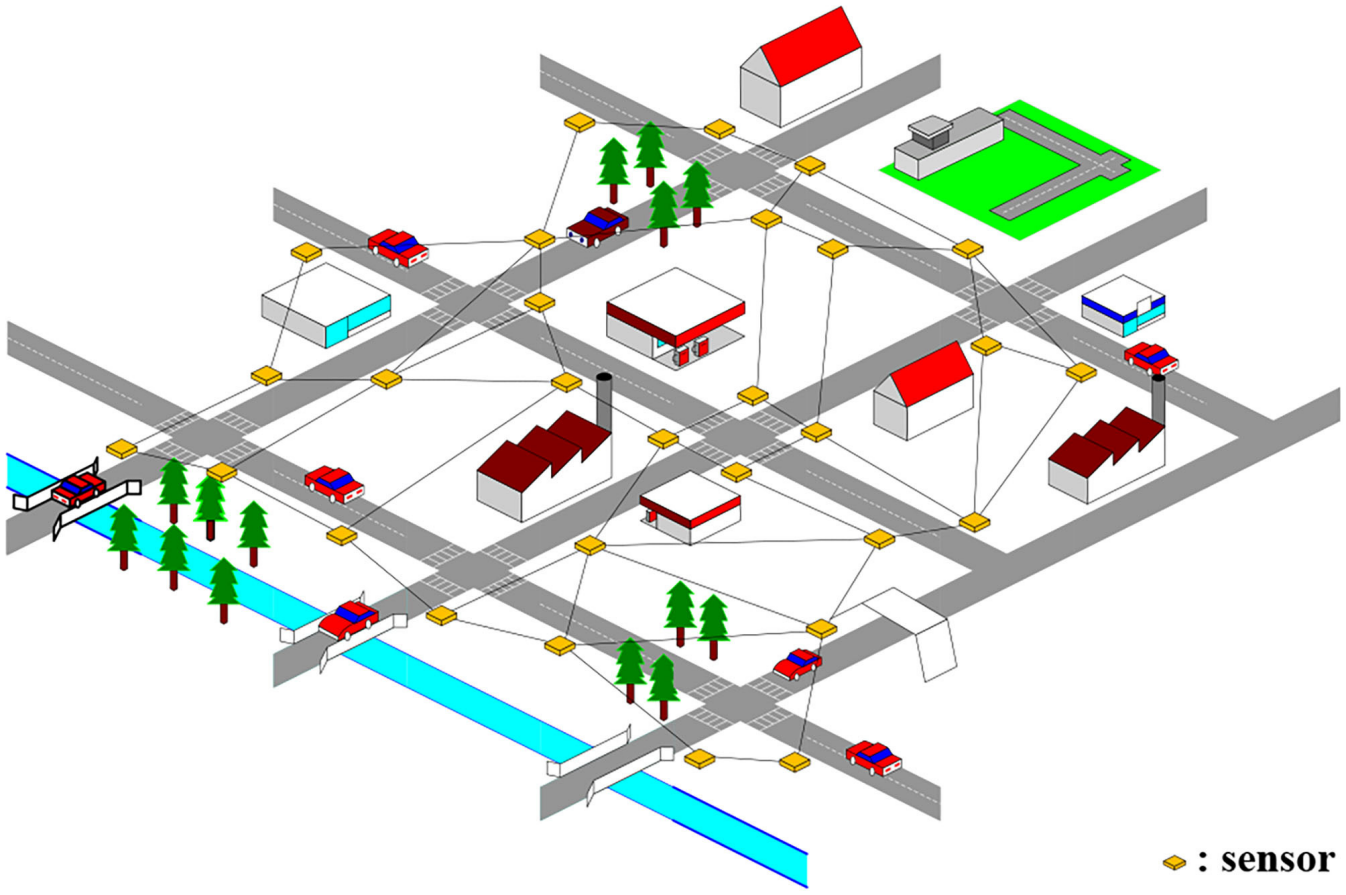
An example of topology graph of traffic network.

As is shown by (Figure 1), the sensors deployed on the traffic network which collected traffic data at fixed time intervals form a non-Euclidean topological graph naturally (34). We use nodes to represent the locations of traffic sensors, and the road segments connecting traffic sensors are treated as edges in graph. After that, the traffic network can be abstracted into a topology graph. Due to the traffic network structure's stability (35), we can keep the structure of its topology graph fixed, which means that V^(τ)^ and E^(τ)^ do not change over time. We define the traffic network at time slice τ as an undirected spatial-temporal graph G^(τ)^ = (X^(τ)^, V, E). Then the traffic spatial-temporal sequence G can be defined as {X^(1)^, X^(2)^, X^(3)^, ⋯ , X^(τ)^, ⋯ , X^(t)^; V, E}, where X^(τ)^ ∈ R^n×c^ denotes the values of all the features of traffic sensors at time slice τ.

### Traffic Flow Forecasting Problem

**Figure 2:**
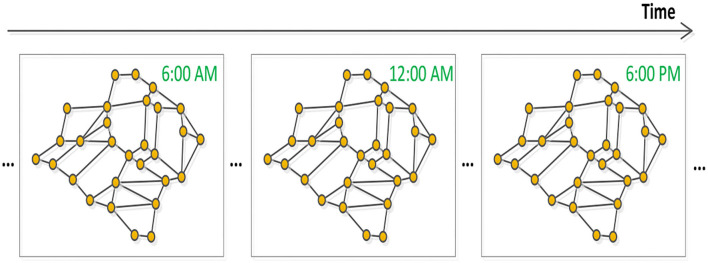
Traffic temporal-spatial graph sequence.

Based on the above analysis, the traffic flow forecasting problem can be defined as a temporal-spatial sequence prediction problem based on graphs, which is shown by (Figure 2).

Given the previous t time slices traffic status X = (X^(1)^, X^(2)^, X^(3)^, ⋯ , X^(τ)^, ⋯ , X^(t)^) ∈ R^n×c×t^ and the graph structure V, E, our task is to predict the future q time slices which can be denoted as:


(1) $$\begin{array}{l} \left\{{\text{X}}^{\left(1\right)},{\text{X}}^{\left(2\right)},\cdots \;,{\text{X}}^{\left(\text{τ}\right)},\cdots \;,{\text{X}}^{\left(\text{t}\right)};\text{V},\text{E}\right\}\overset{\text{MAP}}{\to }\\ \;\left({\text{X}}^{\left(\text{t}+1\right)},{\text{X}}^{\left(\text{t}+2\right)},\cdots \;,{\text{X}}^{\left(\text{t}+\text{q}\right)}\right) \end{array}$$

## Methodology

In this section, we elaborate the framework of our model and its basic modules (the spatial patterns modeling part and the temporal patterns modeling part).

### Network Architecture

**Figure 3:**
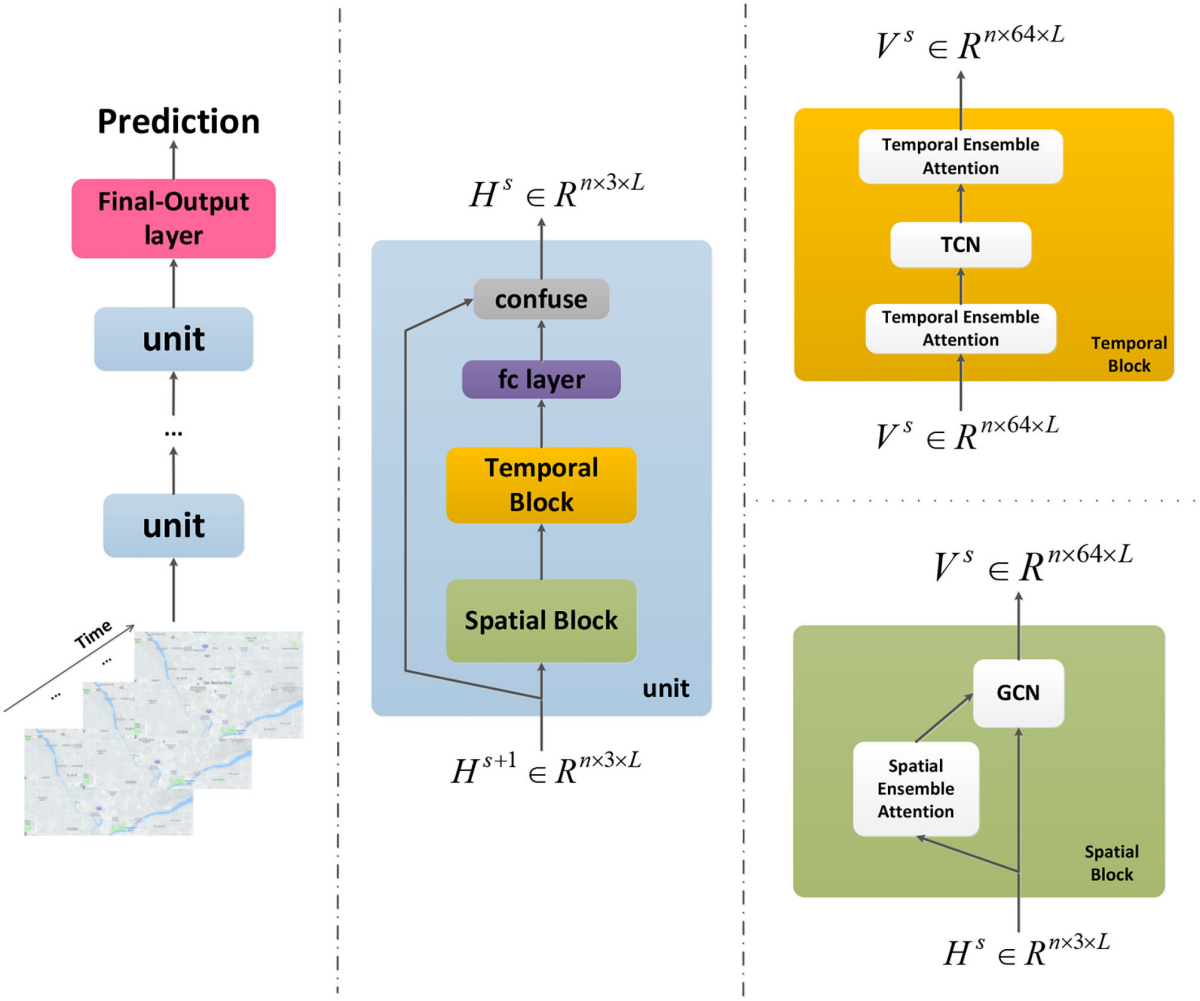
The framework of our model.

The model presented here is an end-to-end framework which is showed by (Figure 3). It has N units and a final-output layer. The final-output layer can generate the final prediction results by integrating comprehensive features. Each unit consists of a spatial block, a temporal block, a fully connected layer and a confuse layer. There is a GCN module and a spatial ensemble attention module in the spatial block. Each temporal block contains two temporal ensemble attention modules and a TCN module in the middle. In the confuse layer, residual connection is applied to optimize the training efficiency and reshape the output of this unit. The dynamic heterogeneous spatial-temporal patterns of the traffic flow are going to be captured elaborately by the overall framework. The main parts of this architecture will be described in details as following sections.

### Spatial Patterns Modeling

#### Graph Convolution

Traffic prediction is a typical task where data are generated from non-Euclidean domains, and the traffic network can be represented as graphs naturally where nodes have complex spatial correlations. Those frequently-used deep learning methods such as CNNs which are born to deal with Euclidean data cannot solve graph-based problems nicely. Hence, in order to model basic spatial dependencies between nodes per time slice of the sequence better, we apply the GCN method.

The GCN method applied here defines convolution filters from the view of signal processing (36) where the convolution operation is treated as removing noises from graph signals. We use the normalized graph Laplacian matrix to represent the graph (36, 37), defined as $\text{L}={\text{I}}_{\text{n}}-{\text{D}}^{-\frac{1}{2}}\text{A}{\text{D}}^{-\frac{1}{2}}\;$, where I_n_ is a n-dimension identity matrix, A ∈ R^n×n^ is the adjacent matrix and D is a diagonal matrix of node degrees (${\text{D}}_{\text{ii}}=\sum_{\text{j}}{\text{A}}_{\text{ij}}$). Due to normalized graph Laplacian matrix's real symmetric positive semidefinite property, it could be decomposed asL = UΛU^T^, where eigenvectors matrix $\text{U}=\left[{\text{u}}_{0},{\text{u}}_{1},{\text{u}}_{2},\cdots \;,{\text{u}}_{\text{n}-1}\right]\in {\text{R}}^{\text{n}\times \text{n}}$ forms an orthonormal space and Λis the matrix of eigenvalues (Λ_ii_ = λ_i_). On the other hand, ${\text{X}}_{:,:,\text{τ}:\text{τ}+1}=\left[{\text{x}}^{\left(0\right)},{\text{x}}^{\left(1\right)},{\text{x}}^{\left(2\right)},\cdots \;,{\text{x}}^{\left(\text{i}\right)},\cdots \;,{\text{x}}^{\left(\text{N}-1\right)}\right]\in {\text{R}}^{\text{n}\times 3\times 1}$ represents all the signals of the graph in time slice τ, where x^(i)^ is the signal value of i_th_ node. Based on U from the decomposition of L, we can define the graph Fourier transformation to X_:, :, τ:τ+1_ as $\hat{{\text{X}}_{:,:,\text{τ}:\text{τ}+1}}=\text{F}\left({\text{X}}_{:,:,\text{τ}:\text{τ}+1}\right)={\text{U}}^{\text{T}}{\text{X}}_{:,:,\text{τ}:\text{τ}+1}$ and the inverse graph Fourier transformation can be defined as ${\text{F}}^{-1}\left(\hat{{\text{X}}_{:,:,\text{τ}:\text{τ}+1}}\right)=\text{U}\hat{{\text{X}}_{:,:,\text{τ}:\text{τ}+1}}$, where $\hat{{\text{X}}_{:,:,\text{τ}:\text{τ}+1}}$ is the signal after graph Fourier transformation. The graph Fourier transformation is able to reflect the graph signal X_:, :, τ:τ+1_ to an orthonormal space built by eigenvectors matrix U.

Based on this, we can define the graph convolution operation as:


(2) $$\begin{array}{l} {\text{X}}_{:,:,\text{τ}:\text{τ}+1}{\;}^{*}\text{G g}={\text{F}}^{-1}\left(\text{F}\left({\text{X}}_{:,:,\text{τ}:\text{τ}+1}\right)⊙\text{F}\left(\text{g}\right)\right)\\ \;=\text{ U}\left({\text{U}}^{\text{T}}{\text{X}}_{:,:,\text{τ}:\text{τ}+1}⊙{\text{U}}^{\text{T}}\text{g}\right) \end{array}$$

where ${\;}_{\text{G}}^{*}$ is the graph convolution operation, and ⊙ denotes the element-wise product. We usually treat diag(U^T^*g*) as the graph convolution filter *g*_θ_ which is made up of some learnable parameters. Then the formula (1) could be simplified as below:


(3) $$\begin{array}{l} {\text{X}}_{:,:,\text{τ}:\text{τ}+1}{\;}^{*}\text{G g}=\text{U}{\text{g}}_{\theta }{\text{U}}^{\text{T}}{\text{X}}_{:,:,\text{τ}:\text{τ}+1} \end{array}$$

However, the eigen-decomposition of the Laplacian matrix requires O(n^3^) computational complexity, which brings too much computation cost. Therefore, Chebyshev polynomials are applied to reduce the computation expense of the graph convolution operations (38). We use the Chebyshev polynomials of the diagonal matrix of eigenvalues (30) to approximate the filter ${\text{g}}_{\theta }=\sum_{\text{i}=0}^{\text{k}}\theta_{\text{i}}{\text{T}}_{\text{i}}\left(\tilde{\text{Λ}}\right)$, where $\tilde{\text{Λ}}=\text{Λ}/{\text{λ}}_{\max}-{\text{I}}_{\text{n}}$, $\theta_{\text{i}}\in {\text{R}}^{\text{k}}$ denotes the co-efficient and λ_max_ is the maximum eigenvalue of the Laplacian matrix. The Chebyshev polynomials are defined recursively by T_i_(φ) = 2φT_i−1_(φ) − T_i−2_(φ) with T_0_(φ) = 1 and T_1_(φ) = φ. As a result, the graph convolution of signal X_:, :, τ:τ+1_ with the defined filter g_θ_ can finally be defined as:


(4) $$\begin{array}{l} {\text{X}}_{:,:,\text{τ}:\text{τ}+1}{\;}^{*}\text{G g}=\text{U}\left(\overset{k}{\underset{i=0}{\sum }}\theta_{\text{i}}T_{\text{i}}\left(\tilde{\Lambda }\right)\right)U^{\text{T}}X_{:,:,\tau :\tau +1}\\ \;=\left(\overset{k}{\underset{i=0}{\sum }}\theta_{\text{i}}T_{i}\left(\tilde{\text{L}}\right)\right){\text{X}}_{:,:,\text{τ}:\text{τ}+1} \end{array}$$

where $\tilde{\text{L}}=2\text{L}/\lambda_{\max}-{\text{I}}_{\text{n}}$.

#### Spatial Ensemble Attention

In the traffic networks, the traffic conditions of one node exert significant but different influence on others'. This kind of traffic heterogeneous spatial correlations can be reflected in the traffic speed, the traffic flow and the traffic density. According to the traffic flow theory, there is a complex relation among them (39), and a multitude of models describing relationships between traffic flow and the other two variables have been developed over the years (40–43). We are supposed to ensemble those factors together to get more comprehensive spatial correlations among nodes, rather than relying solely on the traffic flow.

**Figure 4:**
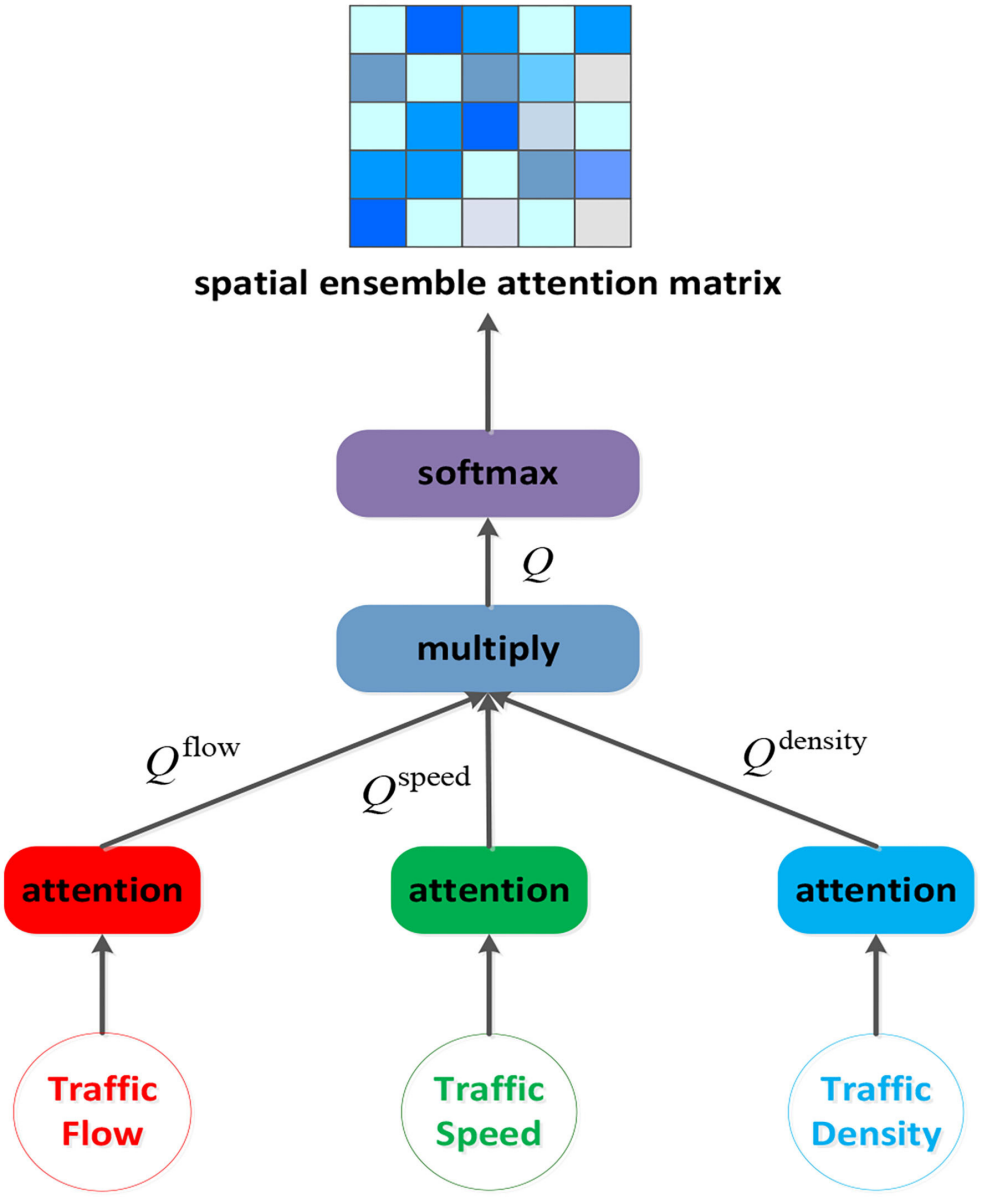
The structure of spatial ensemble attention layer.

Considering of that, a spatial ensemble attention mechanism is proposed which aims to dig out richer dynamic heterogeneous relationships between nodes by packing each factor's attention matrix together.


(5) $$\begin{array}{l} {\text{Q}}^{\left(\text{c}\right)}=\;{\text{V}}_{\text{s}}^{\left(\text{c}\right)}\sigma \left(\left({\text{X}}_{:,\text{c}:\text{c}+1,:}{\text{W}}_{1}^{\left(\text{c}\right)}\right){\text{W}}_{2}^{\left(\text{c}\right)}+{\text{b}}_{\text{s}}^{\left(\text{c}\right)}\right) \end{array}$$

(6) $$\begin{array}{l} \text{Q}=\overset{2}{\underset{\text{c}=0}{\prod }}{\text{Q}}^{\left(\text{c}\right)} \end{array}$$

(7) $$\begin{array}{l} {\text{Q}}_{{\text{i},\text{j}}^{\prime }}=\text{softmax}\left({\text{Q}}_{\text{i},\text{j}}\right) \end{array}$$

where ${\text{X}}_{:,\text{c}:\text{c}+1,:}\in {\text{R}}^{\text{n}\times 1\times \text{L}}$ denotes one feature slice from the spatial ensemble attention module's input ${\text{X}}_{:,;,:}\in {\text{R}}^{\text{n}\times 3\times \text{L}}$, where *n* is the number of nodes, three is the number of input graph signal's features (the traffic flow, the traffic speed and the traffic density), and L is the length of the time length. ${\text{V}}_{\text{s}}^{\left(\text{c}\right)}\in {\text{R}}^{\text{n}\times \text{n}}$, ${\text{b}}_{\text{s}}^{\left(\text{c}\right)}\in {\text{R}}^{\text{n}\times \text{n}}$, ${\text{W}}_{1}^{\left(\text{c}\right)}\in {\text{R}}^{\text{L}}$ and ${\text{W}}_{2}^{\left(\text{c}\right)}\in {\text{R}}^{\text{L}\times \text{n}}$ are the learnable parameters. We construct the spatial ensemble attention matrix Q ∈ R^n×n^ by multiplying the three factors' attention matrix Q^(c)^. After the ensemble operation, the softmax function is used to make sure the spatial ensemble attention matrix's elements sum to one. The spatial ensemble attention operation is presented by (Figure 4).

After this, we accompany the Chebyshev polynomials filters with the spatial ensemble attention matrix Q′. Then the s_th_ spatial block's output can be obtained by:


(8) $$\begin{array}{l} {\text{V}}_{:,:,:}^{\text{s}}=\sigma \left(\left(\overset{k}{\underset{m=0}{\sum }}\theta_{\text{m}}T_{\text{m}}\left(\tilde{L}\right)⊙{\text{Q}}^{\text{′}}\right)H_{:,0:1,:}^{s}\right) \end{array}$$

where s is the index of the spatial block and the σ function is Rectified Linear Unit (ReLU). ${\text{H}}_{:,0:1,:}^{\text{s}}\in {\text{R}}^{\text{n}\times 1\times \text{L}}$ is the traffic flow slice of the s_th_ spatial block's input ${\text{H}}_{:,:,:}^{\text{s}}\in {\text{R}}^{\text{n}\times 3\times \text{L}}$, and ${\text{V}}_{:,:,:}^{\text{s}}$ denotes the s_th_ spatial block's output signal.

### Temporal Patterns Modeling

#### Time Convolution

In the temporal trend analysis, the RNN-based methods are applied extensively. However, the recurrent networks are still stuck in some problems, such as exploding/vanishing gradients and time-consuming iterations (44). On the other hand, the traditional 1D convolution method does not have enough ability to memorize long historical information.

**Figure 5:**
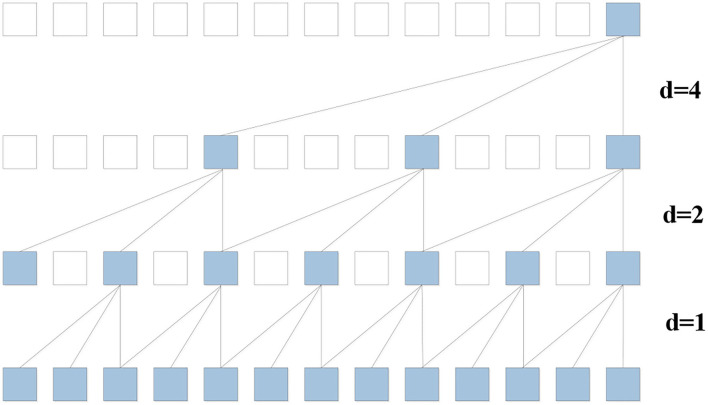
Time convolution net with kernel size three and dilation factor *d* = 1, 2, 4.

Considering the issues above, the TCN frame (45), a simple but highly effective network, is employed to capture the basic temporal dependencies of traffic flow. As is showed in Figure 5, this special designed network supports parallel training procedures to improve training efficiency. Meanwhile, this simple but effective network has ability to look far enough into the past to improve prediction accuracy.

The convolution operation in TCN is dilated causal convolution, which is a variant of causal convolution. Suppose input X_v:(v+1), :, :_ is a traffic flow temporal sequence at the node v and there is a filter f:{0, 1, 2, ⋯ , k − 1}. The dilated causal convolution operation on the u element of the sequence can be defined as:


(9) $$\begin{array}{l} \left({\text{X}}_{\text{v}:\left(\text{v}+1\right),:,:}{\;}^{{*}_{\text{d}}\text{f}}\right)\left(\text{u}\right)=\overset{\text{k}-1}{\underset{\text{i}=0}{\sum }}\text{f}\left(\text{i}\right){\text{X}}_{\text{v}:\left(\text{v}+1\right),:,:}\left(\text{u}-\text{id}\right) \end{array}$$

where u − id accounts for the direction of the past and d is the dilation factor. The dilation factor d is the key parameter to control the distance between every two adjacent filter taps. When the dilation factor is set to one, the dilation causal convolution reduces to the casual convolution. We increase the dilation factor d exponentially with the depth of the network, and the receptive field of the model grows exponentially. This ensures that the long effective historical information can always be captured by some filters.

#### Temporal Ensemble Attention

**Figure 6:**
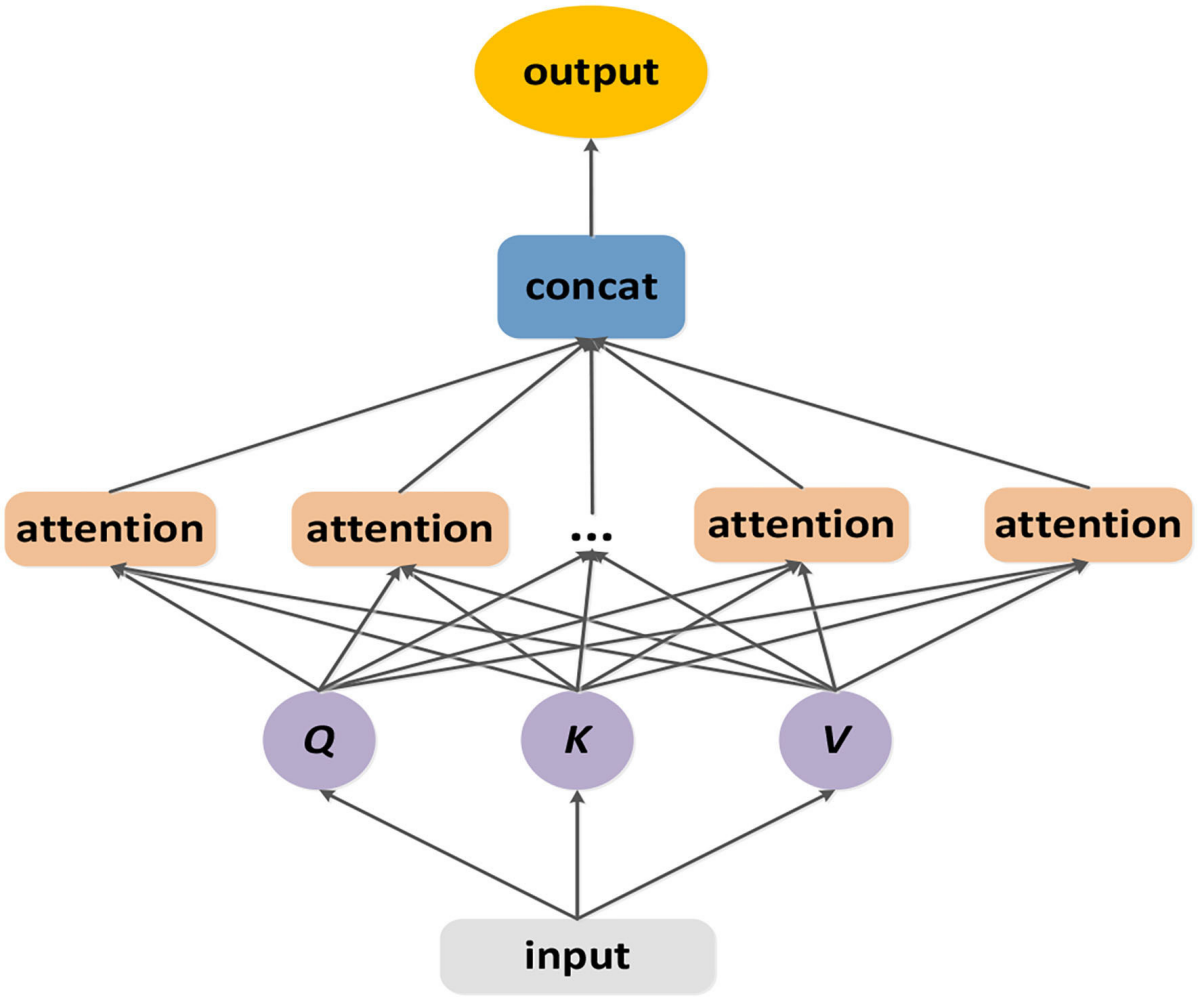
The structure of temporal ensemble attention layer.

In the temporal dimension, the previous traffic conditions have significant but different influence on the following conditions, too. And the correlations among different time slices are complex. Motivated by the transformer framework's multi-head attention (46), we designed a temporal ensemble attention mechanism which is showed by (Figure 6). The temporal ensemble attention operation can capture more comprehensive heterogeneous temporal correlations by expanding bigger feature space at a less computation cost.

The temporal ensemble attention operation is designed based on the self-attention mechanism.


(10) $$\begin{array}{l} \text{SA}=\text{selfattention}\left(\text{Q},\text{K},\text{V}\right)=\text{softmax}(\frac{{\text{QK}}^{\text{T}}}{\sqrt{\text{C}}})(\text{V}) \end{array}$$

where the Q ∈ R^n×L×c^ is the query matrix, the K ∈ R^n×L×c^ is the key matrix and the V ∈ R^n×L×c^ is the value matrix. The matrixes Q, K, V correspond with the input of the temporal ensemble attention module. The output SA is computed as a weight sum of the value matrix, where the weight assigned to each element is obtained by computing the compatibility of the query with the corresponding key.

Due to the complex relationships among the time slices, there is a need to expand the feature space to represent the traffic status. But too many features would increase computational expense. Learning from the multi-head attention (46), we linearly project the query, key and value h times. On each of these projected versions of query, key and value, the self-attention operation is applied in parallel. We contact those outputs and project it linearly to get the final outcome.


(11) $$\begin{array}{l} {\text{Q}}_{\text{i}}={\text{QW}}_{\text{i}}^{\text{Q}} \end{array}$$

(12) $$\begin{array}{l} {\text{K}}_{\text{i}}={\text{KW}}_{\text{i}}^{\text{K}} \end{array}$$

(13) $$\begin{array}{l} {\text{V}}_{\text{i}}={\text{VW}}_{\text{i}}^{\text{V}} \end{array}$$

(14) $$\begin{array}{l} \text{S}{\text{A}}_{\text{i}}=\text{selfattention}\left({\text{Q}}_{\text{i}},{\text{K}}_{\text{i}},{\text{V}}_{\text{i}}\right)\\ \;=\text{softmax}(\frac{{\text{Q}}_{\text{i}}{\text{K}}_{\text{i}}^{\text{T}}}{\sqrt{{\text{C}}_{\text{k}}}})\left({\text{V}}_{\text{i}}\right) \end{array}$$

(15) $$\begin{array}{l} \text{TEA}\left(\text{Q},\text{K},\text{V}\right)=\text{Concat}\left(\text{S}{\text{A}}_{1},\text{S}{\text{A}}_{2},\text{S}{\text{A}}_{3}\cdots \;,\text{S}{\text{A}}_{\text{i}},\cdots \;,\text{S}{\text{A}}_{\text{h}}\right)\\ \;{\text{W}}^{\text{o}} \end{array}$$

where ${\text{W}}_{\text{i}}^{\text{Q}}\in {\text{R}}^{\text{c}\times {\text{C}}_{\text{k}}}$, ${\text{W}}_{\text{i}}^{\text{K}}\in {\text{R}}^{\text{c}\times {\text{C}}_{\text{k}}}$, ${\text{W}}_{\text{i}}^{\text{V}}\in {\text{R}}^{\text{c}\times {\text{C}}_{\text{k}}}$, C_K_ = c/h, W^o^ ∈ R^c×c^. The temporal ensemble attention can not only confuse information from subspace but also balance the conflicts between model's express ability and the computation cost.

## Experiments

In this section, in order to evaluate the performance of our model, we verify it on two publicly-available traffic datasets, showing that our model outperforms the baselines, especially in the long-term forecasting situation.

### Datasets and Preprocessing

We validate our model on two highway traffic datasets: PeMSD-08 and PeMSD-04 (12). The datasets were collected by a system called Caltrans Performance Measurement System (PeMS). On the highways of major metropolitan areas in California, more than 39,000 detectors were applied in this system to collect geographic and traffic information about the sensor locations. PeMSD-08 was collected in San Bernardino from July to August in 2016. This dataset contains 170 detectors on eight roads where the distance between any adjacent detectors is longer than 3.5 miles. The traffic data were aggregated every 5 min, so each detector contains 288 data points per day. PeMSD-04 is the traffic dataset collected by 307 detectors on 29 roads in San Francisco Bay Area. In this dataset, the time range is from January to February in 2018, and the time interval between two data points is 5 min, too.

To improve the model's efficiency and performance, it is necessary to normalize the input data and map their attribute values between[0, 1]. Min–max normalization was used to preprocess the datasets.


(16) $$\begin{array}{l} {\text{x}}^{\prime }=\frac{\text{x}-{\text{x}}_{\min}}{{\text{x}}_{\max}-{\text{x}}_{\min}} \end{array}$$

where *x*_min_ denotes the minimum value of the input data, *x*_max_ denotes the maximum value of the input data, x is the observed data and *x*′ is the normalized data.

We build the weighted adjacency matrix by road network distance:


(17) $$\begin{array}{l} {\text{A}}_{\text{ij}}=\{\begin{array}{c} 1\text{ if dist}\left({\text{V}}_{\text{i}},{\text{V}}_{\text{j}}\right)\text{>}E\\ 0\text{ otherwise} \end{array} \end{array}$$

where A_ij_ represents the edge between node i and node j, *dist*(*V*_*i*_, *V*_*j*_) represents the distance between node i and node j, and is the threshold to control the distribution and sparsity of matrix A.

### Settings

We split the datasets in chronological order with the first 60% for training, next 20% for testing and the remaining 20% for validation. The proposed architecture is implemented by PyTorch (1.3.1 version) and trained on a computer with NVIDIA GeForce GTX 1050 GPU and Intel(R) i5-6500 CPU. The data input length is set to 24. Adam optimizer is chosen to optimize the parameters of our deep learning framework.

In our model, we set Chebyshev polynomial *K* = 3. As K continues to increase, the model's performance improves slightly with a much higher computational cost. The feature of GCN network's output is set to 64. The number of TCN's layers is set to four. The kernel size in TCN is set to three. We set the dilation d = 2^l^, where l ∈ {0, 1, 2} is the index number of layers in TCN. The number of the sub-attention block in temporal ensemble attention module is set to four. During the training phase, the batch size is eight and the learning rate is 0.001.

Two commonly used metrics: Mean Absolute Errors (MAE) and Root Mean Squared Errors (RMSE) were selected to evaluate the performance of different models.


(18) $$\begin{array}{l} \text{MAE}\left(y,ŷ\right)=\frac{1}{Q}\overset{Q}{\underset{i=1}{\sum }}|y_{\text{i}}-\hat{y_{i}}| \end{array}$$

(19) $$\begin{array}{l} \text{RMSE}\left(y,ŷ\right)=\sqrt{\frac{1}{Q}\overset{Q}{\underset{i=1}{\sum }}{\left(y_{i}-\hat{y_{i}}\right)}^{2}} \end{array}$$

where Q denotes the size of the testing dataset, y denotes the ground truth and $\hat{\text{y}}$ denotes the predicted value.

### Baselines

We compare our model with several baseline models, including traditional machine learning methods (SVR, RF) and recently published state-of-the-art deep learning models (GRU, LSTM, Seq2Seq, ASTGCN) in traffic forecasting domains.

➢ SVR: Support Vector Regression, which uses support vector machine for the regression task. The model was implemented based on scikit-learn python package. The penalty term was set as 0.1, kernel type was set as “rbf,” and the number of historical observation was set as 24.
➢ RF: Random Forest, which is made of many decision trees. The model was implemented based on scikit-learn python package. Estimators were set as 100, random state was set as 42, and the number of max features was set as six.
➢ GRU: Gated Recurrent Unit network, which is a special RNN model (47). The hidden size of GRU is set as 64.
➢ LSTM: Long Short Term Memory network, which is a special RNN model (48). The hidden size of LSTM is set as 64.
➢ Seq2Seq: Sequence to Sequence model, which is composed of the encoder and the decoder. The hidden size of both encoder and decoder GRUs are set as 64.
➢ ASTGCN: Attention Based Spatial-Temporal Graph Convolutional Network, which is composed of graph convolutional layers and normal 1-D convolutional (12).

### Performance Comparisons and Analysis

Table 1 shows the performance of our model and other baselines for the traffic flow predictions in the next 15, 30, and 60 min on PeMSD-08 dataset.

**Table 1:** Traffic flow forecasting comparison in the next 15, 30 and 60 min on PeMSD08.

**Models**	**MAE**			**RMSE**		
	**15 min**	**30 min**	**60 min**	**15 min**	**30 min**	**60 min**
SVR	18.98	19.34	23.96	30.25	27.80	34.17
RF	19.52	20.02	24.26	30.34	28.25	33.97
GRU	21.51	23.70	26.14	31.66	34.51	37.39
LSTM	20.20	20.75	23.14	29.40	30.28	34.28
Seq2Seq	22.12	22.81	24.72	34.96	35.93	38.38
ASTGCN	17.32	18.65	21.77	25.30	27.52	31.68
Ours	16.59	17.58	18.96	24.28	26.89	28.72
Improvement	2.14%	5.74%	12.91%	4.03%	5.19%	9.34%

It's obvious that our model obtains the best results in terms of all evaluation metrics. Specially, it is worth noting that the improvement of our model's performance than the second-best model (ASTGCN) increase as the forecasting horizon grows longer, which is shown in the last row of Table 1.

**Figure 7:**
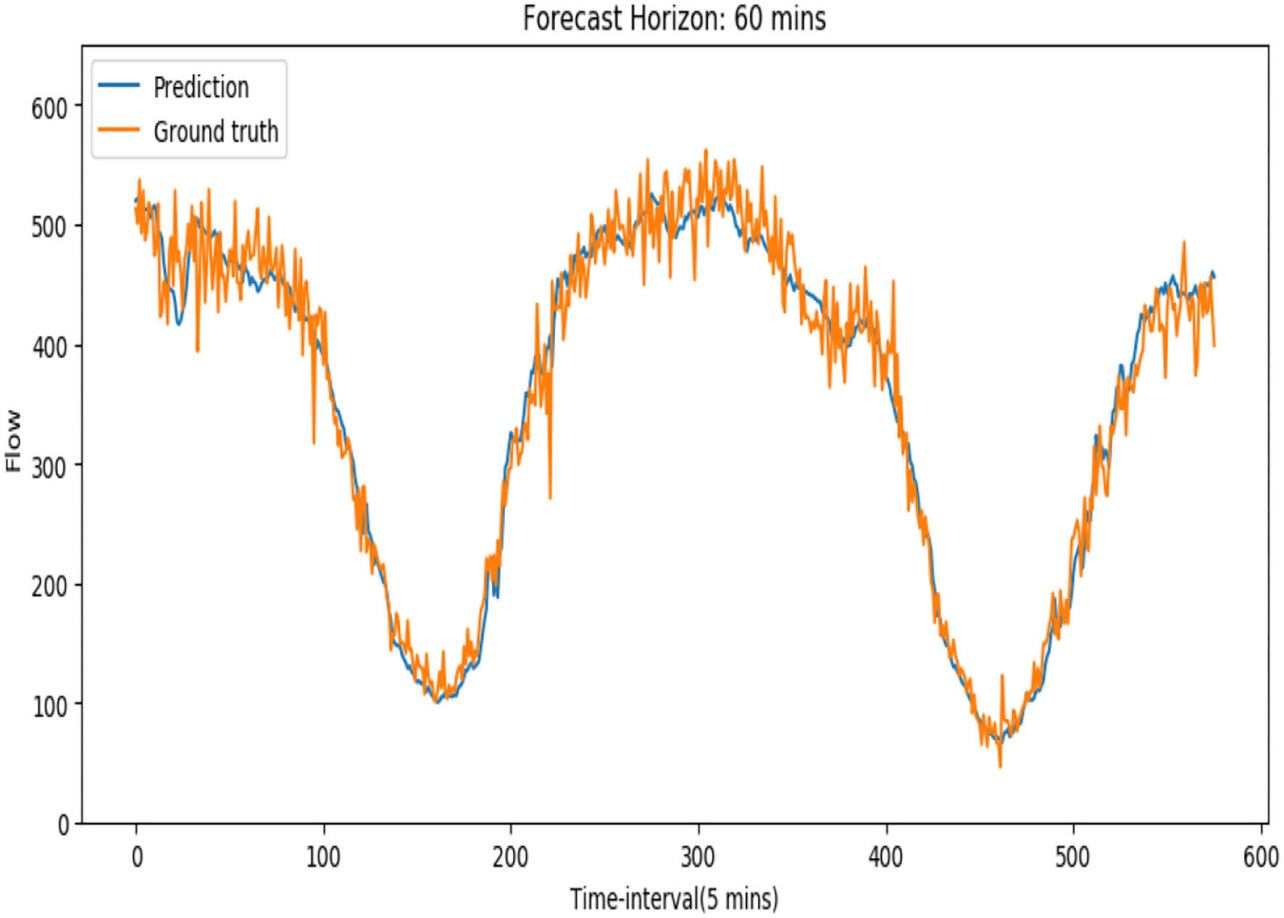
Traffic flow prediction and the corresponding ground truth of a sensor during 2 days.

To further verify the superiority of our model in the long horizon forecasting situation, we compared the performances of our model and the other baselines for the next 60 min traffic flow prediction on both PeMSD-08 and PeMSD-04 datasets, which is shown by (Table 2). Obviously in the long-term traffic flow prediction situation, our model does perform better. We think this is because the TCN network in our model has stronger ability to capture long historical information. And the normal 1-D time convolution in ASTGCN model cannot get enough information from the past. On the other hand, Table 3 shows two models' computation cost in the training. Our model costs less training time than the ASTGCN model. Figure 7 shows the 60-min-ahead predicted values and the ground truth of a certain sensor in 2 days.

**Table 2:** Traffic flow forecasting comparison in the next 60 min on PeMSD04 and PeMSD08.

**Data**	**Models**	**60 min**	
		**MAE**	**RMSE**
PEMS-08	SVR	23.96	34.17
	Random Forest	24.26	33.97
	GRU	26.14	37.39
	LSTM	23.14	34.28
	Seq2Seq	24.72	38.38
	ASTGCN	21.77	31.68
	Ours	18.96	28.72
PEMS-04	SVR	29.49	41.49
	Random Forest	29.57	41.28
	GRU	28.79	44.02
	LSTM	26.59	40.36
	Seq2Seq	26.49	42.08
	ASTGCN	26.00	41.12
	Ours	25.51	37.15

**Table 3:** Training efficiency comparison.

**Model**	**Average training time (s/epoch)**
ASTGCN	329.84
Our Model	256.07

**Figure 8:**
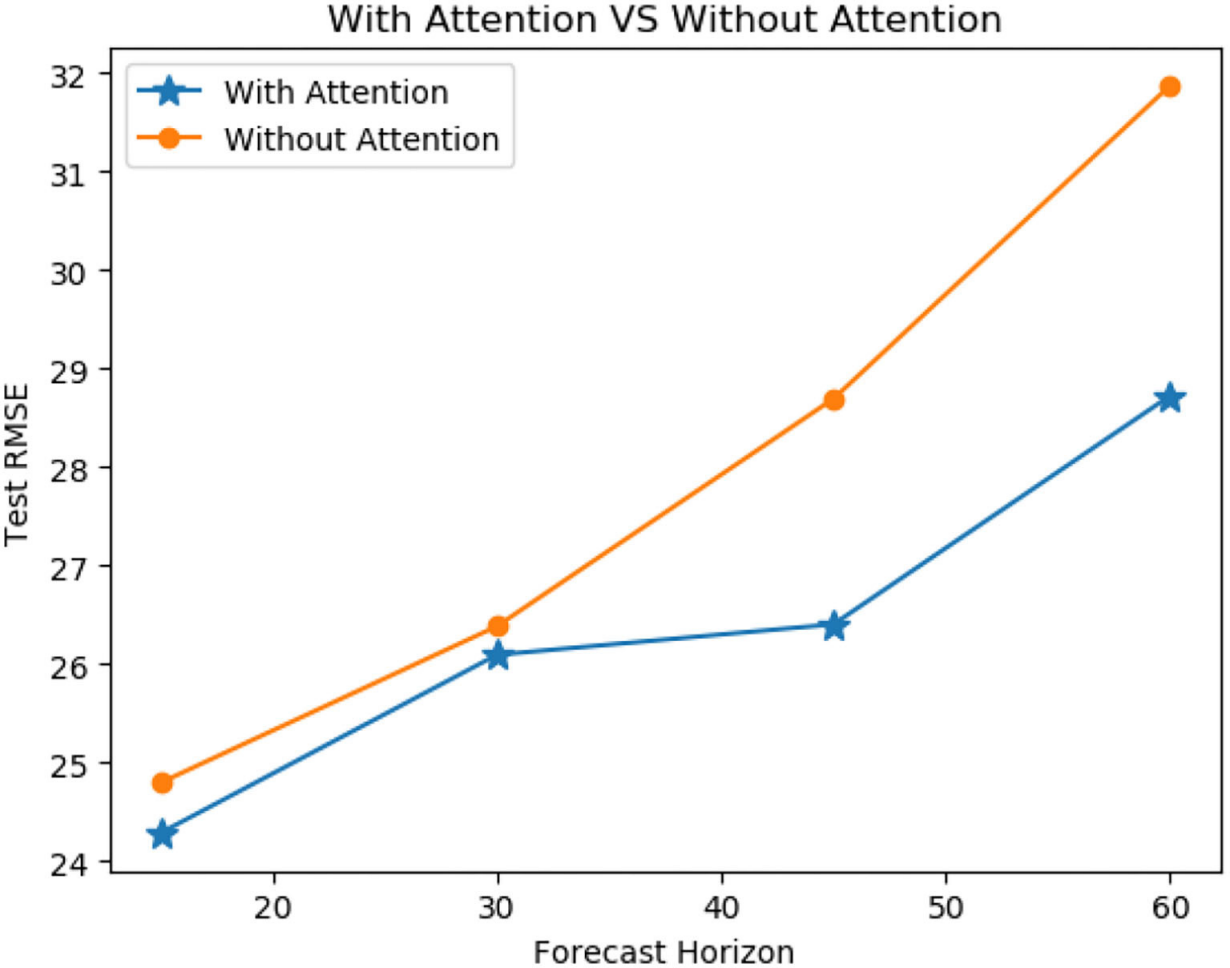
Average performance comparison of models with or without attention mechanism on PeMSD8.

In order to verify the effectiveness of our model's ensemble-attention mechanism, we construct a new model by getting rid of the ensemble attention operation from our model. The reconstructed version of our model can be treated as a simple model stacked of the GCN layer and the TCN layer. We evaluated our model and the reconstructed version for the traffic flow predictions in the next 15, 30, 45, and 60 min on PeMSD-08 dataset. Figure 8 shows the RMSE results of the comparison, and the model with the ensemble-attention mechanism outperforms the reconstructed version especially in the long-term forecasting situation. This means that the ensemble-attention mechanism does play a role in the traffic flow prediction.

Furthermore, we plot a series of heat maps with different color depth to visualize the learned ensemble-attention matrixes. Both the spatial ensemble attention and the temporal ensemble attention are obtained from the last unit of our model.

**Figure 9:**
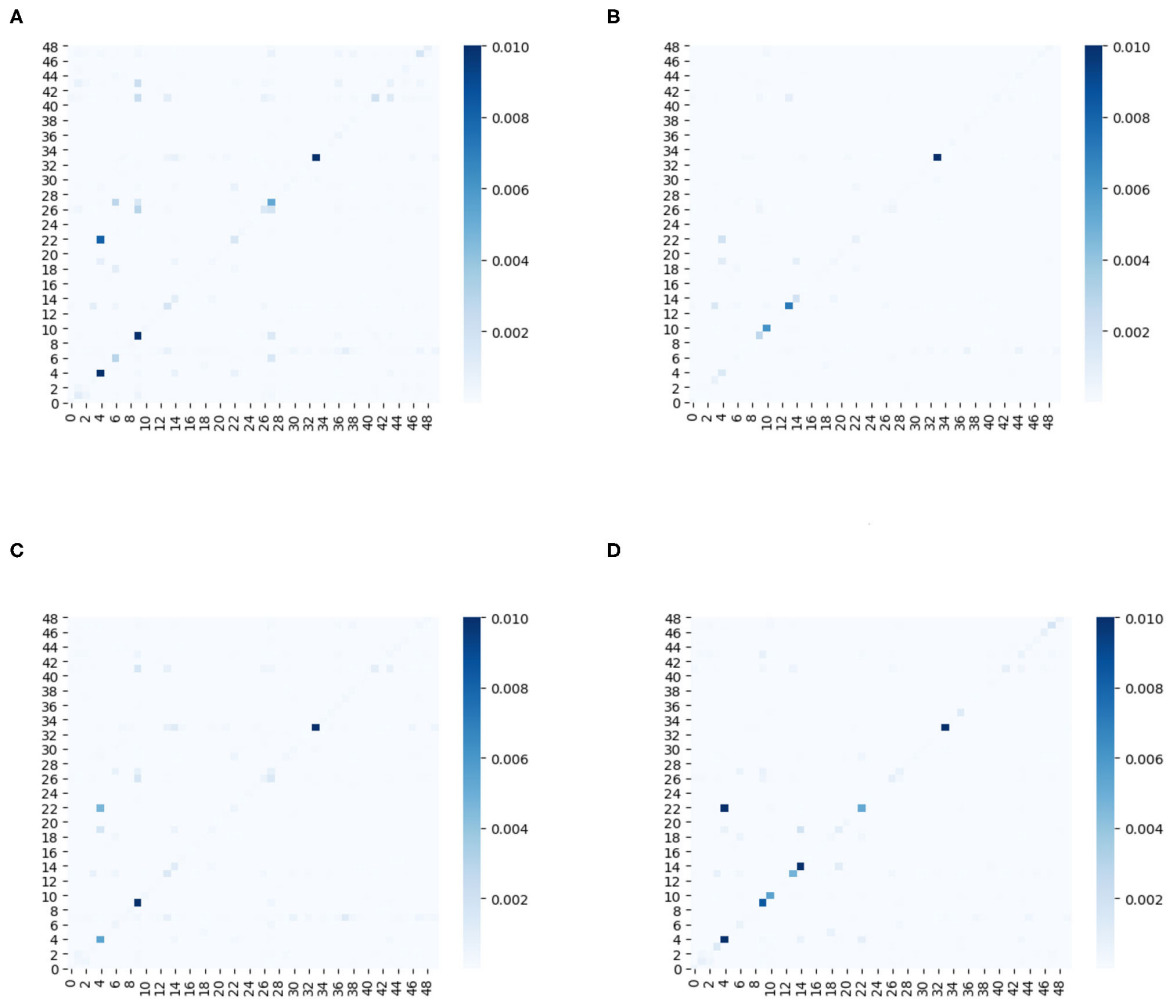
**(A–D)** The heat maps of adjacency matrixes for the first 50 sensors at four different time slices.

On the one hand, the first 50 nodes are picked out to show the spatial correlations among those nodes at four different time slices. Figure 9 shows the heat maps of spatial ensemble attention matrixes at four different time slices. The X-axis and Y-axis denote the 50 detectors. The value of the pixel at point (x, y) is the coefficient of detector y to detector x. The color depth of pixel at point (x, y) indicates the degree of influence that the detector y exerts to the detector x. The pixel with a deep color indicates that the detector x is affected strongly by the detector y. From Figure 9, we can observe that the spatial ensemble attention mechanism does capture dynamic heterogeneous spatial correlations among nodes to a certain extent.

**Figure 10:**
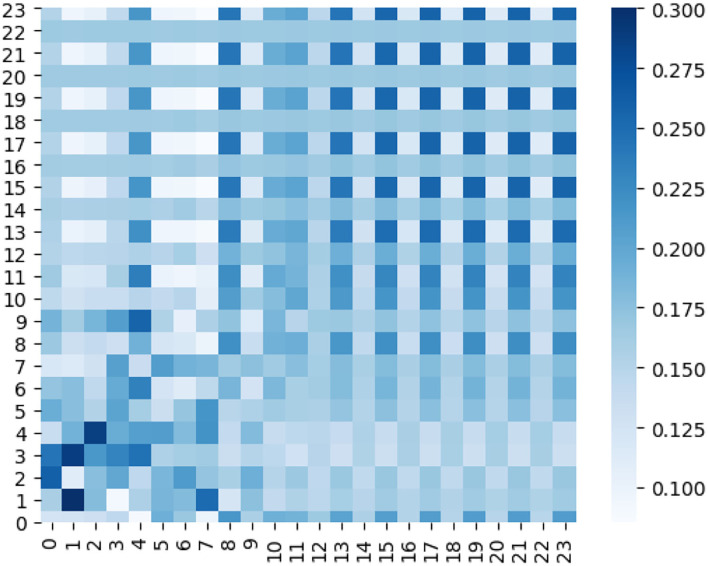
Temporal ensemble attention coefficients of a certain sensor are visualized as a heat map.

**Figure 11:**
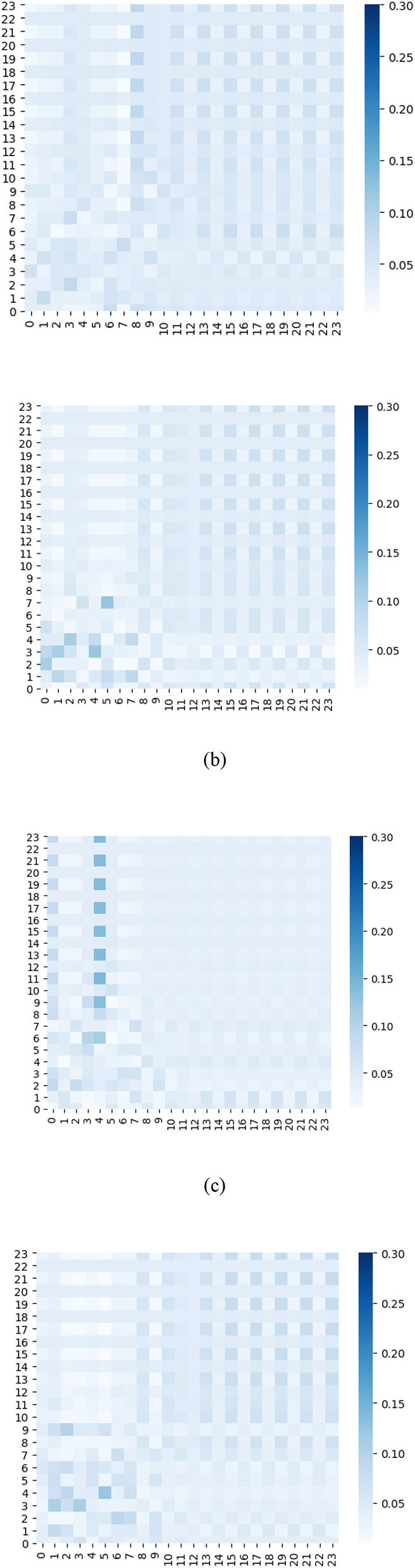
Temporal sub-attention coefficients of time slices are visualized as heat maps.

On the other hand, the heat map of temporal ensemble attention is shown by (Figure 10), and the temporal sub-attention blocks' heat maps are represented by (Figure 11). The X-axis and Y-axis denote the 24 time slices at a sensor node. The value of the pixel at point (x, y) is the coefficient of time slice y to time slice x. The pixel with a deep color indicates that time slice x is affected strongly by the time slice y. From Figures 10, 11, we can observe that the temporal ensemble attention mechanism do capture some heterogeneous temporal correlations.

Based on the above analysis, the ensemble attention mechanism including the spatial part and temporal part do learn some beneficial information which enables the model to exploit the dynamic heterogeneous traffic patterns for traffic forecasting.

## Conclusions and Future Work

In this paper, a novel end-to-end deep learning framework is proposed for traffic flow predicting. The knowledge gained from our research can provide many valuable applications for vehicle emission warnings, improving urban traffic construction and studying the sources of air pollution. Each unit of the models mainly is composed by a spatial block and a temporal block. In the spatial block, we fuse GCN and spatial ensemble attention mechanism to capture global dynamic heterogeneous spatial patterns. In the temporal block, TCN and temporal ensemble attention mechanism are combined to capture non-stationary temporal patterns. The model is fed with a variety of explanatory variables including the historical traffic speed, the historical traffic density, the historical traffic flow and the graph of sensor network. Experiment results show that the forecasting accuracy of the proposed model is superior to existing models especially in the long-term predicting situation and the model can be trained faster than the main DL baselines. The ensemble attention mechanism is shown to be capable of capturing comprehensive dynamic heterogeneous spatial-temporal correlations of traffic series.

Actually, the urban traffic flow is affected by many external factors, such as weather and social events. In the future, we will fuse more external factors into our model to make the predictions of attributes related to traffic emissions more accurate.

## Data Availability Statement

The original contributions presented in the study are included in the article/supplementary material, further inquiries can be directed to the corresponding author.

## Author Contributions

BY: supervision and writing–review and editing. AM: conceptualization, methodology, and writing-original draft. RF: methodology, programming, and writing-improvement. XS: data collection and data analysis. MZ and YY: investigation and data collection. All authors contributed to the article and approved the submitted version.

## Funding

The work described in this paper was jointly supported by grants from National Natural Science Foundation of China (U1811463 and 51675077), the State Key Laboratory of Structural Analysis for Industrial Equipment (S18307).

## Conflict of Interest

XS was employed by CIECC Overseas Consulting Co., Ltd. The remaining authors declare that the research was conducted in the absence of any commercial or financial relationships that could be construed as a potential conflict of interest.

## Publisher's Note

All claims expressed in this article are solely those of the authors and do not necessarily represent those of their affiliated organizations, or those of the publisher, the editors and the reviewers. Any product that may be evaluated in this article, or claim that may be made by its manufacturer, is not guaranteed or endorsed by the publisher.
